# Volumetric, relaxometric and diffusometric correlates of psychotic experiences in a non-clinical sample of young adults

**DOI:** 10.1016/j.nicl.2016.09.002

**Published:** 2016-09-04

**Authors:** Mark Drakesmith, Anirban Dutt, Leon Fonville, Stanley Zammit, Abraham Reichenberg, C. John Evans, Philip McGuire, Glyn Lewis, Derek K. Jones, Anthony S. David

**Affiliations:** a Cardiff University Brain Research Imaging Centre (CUBRIC), School of Psychology, Cardiff University, Manidy Road, Cardiff CF24 4HQ, UK; b Neuroscience and Mental Health Research Institute (NMHRI), School of Medicine, Cardiff University, Maindy Road, Cardiff CF24 4HQ, UK; c Department of Psychosis Studies, Institute of Psychiatry, Psychology and Neuroscience, King's College London, DeCrespigny Park, London SE5 8AF, UK; d Centre for Academic Mental Health, School of Social and Community Medicine, University of Bristol, Bristol BS8 2BN, UK; e Department of Psychiatry, Icahn School of Medicine, Mount Sinai Hospital, 1425 Madison Avenue, New York, NY 10029, USA; f Division of Psychiatry, Faculty of Brain Sciences, University College London, Charles Bell House, 67–73 Riding House Street, London W1W 7EJ, UK

**Keywords:** Psychosis, Grey matter, VBM, DTI, MR relaxometry, ALSPAC, Neurodevelopment

## Abstract

**Background:**

Grey matter (GM) abnormalities are robust features of schizophrenia and of people at ultra high-risk for psychosis. However the extent to which neuroanatomical alterations are evident in non-clinical subjects with isolated psychotic experiences is less clear.

**Methods:**

Individuals (mean age 20 years) with (n = 123) or without (n = 125) psychotic experiences (PEs) were identified from a population-based cohort. All underwent T1-weighted structural, diffusion and quantitative T1 relaxometry MRI, to characterise GM macrostructure, microstructure and myelination respectively. Differences in quantitative GM structure were assessed using voxel-based morphometry (VBM). Binary and ordinal models of PEs were tested. Correlations between socioeconomic and other risk factors for psychosis with cortical GM measures were also computed.

**Results:**

GM volume in the left supra-marginal gyrus was reduced in individuals with PEs relative to those with no PEs. The greater the severity of PEs, the greater the reduction in T1 relaxation rate (R1) across left temporoparietal and right pre-frontal cortices. In these regions, R1 was positively correlated with maternal education and inversely correlated with general psychopathology.

**Conclusions:**

PEs in non-clinical subjects were associated with regional reductions in grey-matter volume reduction and T1 relaxation rate. The alterations in T1 relaxation rate were also linked to the level of general psychopathology. Follow up of these subjects should clarify whether these alterations predict the later development of an ultra high-risk state or a psychotic disorder.

## Highlights

• Differences in cortical R1 in young adults with psychotic experiences were found.
• Implicates developmental processes related to myelination in psychotic illness.
• The study utilises a large and well-controlled epidemiological cohort.
• A novel approach to mapping microstructural parameters in cortex was employed.

## 1 Introduction

Grey matter (GM) abnormalities have been identified in several structures in schizophrenia patients (Ellison-Wright et al., 2008, Haijma et al., 2013, Honea et al., 2005, Velakoulis et al., 2006, Vita et al., 2006, Wright et al., 2000). The most heavily implicated are the prefrontal, parietal, temporal, anterior cingulate cortices, and hippocampus (Kanaan et al., 2005). GM volume is reduced in chronic versus first episode schizophrenia patients (Meisenzahl et al., 2008) and falls progressively with age(Vita et al., 2012).

In order to delineate structural brain differences that might predispose to psychosis, it is important to examine them without confounding effects of medication or chronic illness. Studies that examine first-episode or drug naive patients overcome such issues to some extent. A number of studies have also focused on high-risk groups: individuals presenting with the At-Risk Mental State (ARMS) or those deemed to be at ultra-high-risk (UHR) for psychosis (Fusar-Poli et al., 2013, Simon et al., 2011). Studies of GM alterations in this group have found macroscopic changes in the cerebral cortex (Borgwardt et al., 2007, Mechelli et al., 2011). A meta-analysis of VBM studies (Fusar-Poli et al., 2011) showed that at-risk groups tend to show reductions in GM volume in many of the regions implicated in schizophrenia, although the changes are generally less severe (Ziermans et al., 2009, Ziermans et al., 2012). Other meta-analyses (Borgwardt et al., 2011, Fusar-Poli et al., 2011) also found that reduced GM volume in prefrontal, cingulate, temporo-parietal, insular and cerebellar regions were significant predictors of transition to full-blown psychosis. More recent, larger and better controlled studies show defined GM structural changes associated with both risk of (Cropley et al., 2016) and transition to psychosis (Cannon et al., 2014, Mechelli et al., 2011, Pantelis et al., 2003, Ziermans et al., 2009). Of particular interest is the North American Prodrome Longitudinal Study which showed that young adults who later transition to psychosis have reduced frontal grey matter compared to those who do not transition (Cannon et al., 2015). Another large cohort, The Philadelphia Neurodevelopmental Cohort, of young adults with psychotic experiences showed reduced grey matter volume in medial temporal regions (Satterthwaite et al., 2016).

While numerous studies have examined GM morphometry in schizophrenia and at-risk groups, other imaging modalities that can provide insights into cortical microstructure have seldom been explored. MRI methods commonly used to quantify myelination and microstructure in white-matter may be used as putative measure of cyto- and myeloarchitecture in cortex (Weiskopf et al., 2015). For example, T1 relaxometry (Homer and Beevers, 1985) is sensitive to myelin content in health and disease and can be used to derive high-resolution cortical myelin maps in vivo (Dinse et al., 2015, Lutti et al., 2013). To our knowledge, no studies have investigated in vivo cortical myelination using MRI in schizophrenia or in at risk-groups. Diffusion tensor imaging (DTI) (Basser et al., 1994), has been applied to investigate changes in cortical microstructure in a number of clinical groups (Kang et al., 2012, Muñoz Maniega et al., 2004, Oreja-Guevara et al., 2005, Rovaris et al., 2006, Vite et al., 2008), Most commonly, mean diffusivity (MD) is used as an inverse measure of cellular density. One recent study (Park et al., 2014) examined diffusivity of frontal and temporal cortices and found it to be higher in schizophrenia patients. To our knowledge, there are no published MRI studies of cortical diffusivity in non-familial at-risk groups.

There are several risk-factors associated with the development of psychosis. These include obstetric factors (Abel et al., 2010, Dalman et al., 2001, Foerster et al., 1991, Lewis, 2002, Rifkin et al., 1994) socioeconomic disadvantage (Byrne et al., 2004, Gallagher et al., 2013, O'Donoghue et al., 2015, Werner et al., 2007), premorbid IQ (David et al., 1997, Woodberry et al., 2008, Zammit et al., 2004), childhood trauma and life stress (Gallagher et al., 2013, Kraan et al., 2015, Morgan and Fisher, 2007, Norman and Malla, 1993) and substance abuse (Kelly, 2000, Koskinen et al., 2009, Moore et al., 2007). The pathogenic processes underlying these factors are unknown and multiple mechanisms are possible. Some may operate through the cyto- and myeloarchitecture of the cerebral cortex.

This study utilises a large epidemiological birth cohort, the Avon Longitudinal Study of Parents and Children (ALSPAC) (Boyd et al., 2013, Golding et al., 2001) cohort, which has rich biological, clinical and psychosocial data to examine the association between GM structure and PEs. Neuroimaging of this cohort has previously shown significant alterations white matter circuitry (Drakesmith et al., 2015, Drakesmith et al., 2016). We tested the hypothesis that abnormalities in GM volume and myeloarchitecture are associated with PEs even in the absence of a clinically identified disorder. This approach has a number of advantages. In particular, it allows the examination of PEs without selection biases and confounding factors such as secondary effects of illness including pharmacological treatment. Furthermore, the cohort has accrued unbiased longitudinal data on a number of developmental and socio-economic variables that can constitute risk factors for psychosis, affording us an opportunity to identify any relationships between them and GM abnormalities that may also be linked to PEs.

## 2 Methods and materials

### 2.1 Subjects

Subjects were recruited from the Avon Longitudinal Study of Parents and Children (ALSPAC) (Boyd et al., 2013, Golding et al., 2001) cohort. The original sample consisted of pregnant women whose expected delivery dates were between April 1991 and December 1992 resulting in the birth of 15,458 foetuses (see Supplementary material, Section 1 for full description of cohort). 4320 subjects from the cohort were assessed for psychotic experiences (PEs) using the Psychotic-like Symptoms (PLIKS) semi-structured interview (Horwood et al., 2008, Zammit et al., 2008), conducted at 17/18 years of age by trained psychologists. Those who were found to have one or more psychotic experience were invited to undergo scanning. The presence of PEs was judged according to clinical criteria of the Schedule for Clinical Assessment in Neuropsychiatry (SCAN) (Wing et al., 1990) and excluded experiences occurring due to waking, falling asleep, fever or drug consumption. PEs were further categorised as ‘suspected’ (n = 44), ‘definite’, (n = 47) and ‘clinical disorder’ (PEs plus functional deterioration and/or help-seeking; n = 32) (Zammit et al., 2013). 126 subjects with PEs were recruited and an equal number of randomly-selected controls, who had completed the same assessments but who were rated as not having had PEs experiences were also scanned. At the time of scanning all subjects were approximately 20 years old (see Table 1 for details).

Informed consent was obtained prior to scanning. Ethical approval was granted by the Cardiff University School of Psychology Ethics Committee and the ALSPAC Ethics and Law Committee. Of the subjects initially scanned, 3 PE subjects and 1 control were unable to complete the full MRI acquisition. While all participants completed the T1-weighted structural scan, the sample sizes for the relaxometry and diffusion MRI scans of the two groups were n = 123 and n = 125, respectively.

### 2.2 Risk factors

A number of candidate variables hypothesized to contribute to psychosis risk were obtained from the ALSPAC variable catalogue (http://www.bris.ac.uk/alspac/researchers/data-access/data-dictionary/). These included age and gender plus 12 other risk-factors: (1) IQ at age 8 estimated from the WISC (Wechsler Intelligence Scale for Children (WISC-III^UK^)) (Golombok and Rust, 1992); (2) current general psychopathology at age 17/18 measured using the computerised Clinical Interview Schedule (revised: CIS-R) (Lewis et al., 1992); (3) parental social class (OPCS, 1991); (4) maternal education; (5) birth weight; (6) resuscitation at birth; (7) stressful life events at age 15/16 measured on the Development and Wellbeing Assessment (DAWBA) questionnaire (Goodman et al., 2000); (8) handedness; (9) tobacco use; (10) alcohol consumed; (11) cannabis consumption (all substance use data gathered at age 17/18); (12) month of birth.

Any missing data points for each subject were estimated using regression imputation (S. F. Buck, 1960) across the entire cohort. Descriptive and inferential statistics of all selected risk factors are detailed in Table 1.

## 3 Imaging

### 3.1 Structural MRI acquisition

All MRI data were acquired at Cardiff University Brain Imaging Research Centre, UK on a 3 T General Electric HDx MRI system (GE Medical Systems, Milwaukee, WI) using an eight-channel receive-only head RF coil. T1-weighted structural images were acquired with a 3D fast spoiled gradient echo (FSPGR) sequence (TR = 7.8 ms, TE = 3.0 ms, flip angle 20°, voxel size = 1 mm^3^ isomorphic).

### 3.2 Relaxometry MRI acquisition and pre-processing

Relaxometry images were acquired using driven equilibrium single pulse observation of T1 with high-speed incorporation of RF field inhomogeneities (DESPOT1-HIFI) (Deoni, 2007). A series of spoiled gradient echo (SPGR) images was acquired with 8 flip angles plus an additional inversion-recovery (IR) SPGR image. All images had TE = 2.11 ms and TR = 4.7 ms, voxel resolution: 1.70 × 1.72 × 1.72 mm. SPGR images were acquired with flip angles of 3°, 4°, 5°, 6°, 7°, 9°, 13° and 18°. For the IR-SPGR acquisition, Inversion time = 450 ms and flip angle = 5°. The DESPOT1 protocol constitutes part of the mCDESPOT protocol (Deoni et al., 2008) which was also collected from this cohort. This method and associated findings are reported in Supplementary material, section 2.

Relaxometry data were pre-processed using FSL v5.0 (Jenkinson et al., 2012). All SPGR/IR-SPGR images were coregistered to each other using a rigid affine transform and skull-stripped (Smith, 2002). Relaxation rate (R1 = 1/T1) maps were derived using the (DESPOT1-HIFI) model (Deoni, 2007), which incorporates correction for B1 field inhomogeneities with in-house code. A synthetic T1-weigted image was computed from the quantitative T1 map with contrast matching that of the FSPGR image. This was used to compute a non-rigid affine transform using a mutual information cost function from the DESPOT space to the FSPGR space. This transform was then applied to the R1 maps so that they are now in the same space as the FSPGR T1-weighted images. Mislocalisation error between the two spaces was quantified to assess any potential impact these might have on subsequent statistical analysis (see Supplementary material, Section 4).

### 3.3 Diffusion acquisition and pre-processing

Diffusion MRI comprising a cardiac-gated diffusion-weighted spin-echo echo-planar imaging sequence was used to acquire high angular resolution diffusion weighted images (HARDI) (Jones et al., 1999). 60 gradient orientations and 6 unweighted (b = 0 s/mm^2^) images were acquired with the following parameters: TE = 87 ms, b = 1200 s/mm^2^, 60 slices, slice thickness = 2.4 mm, FoV = 230 × 230 mm, acquisition matrix = 96 × 96, resulting in data acquired with a 2.4 × 2.4 × 2.4 mm isotropic resolution following zero-filling to a 128 × 128 in-plane matrix for the fast Fourier transform. The final image resolution was therefore 1.8 × 1.8 × 2.4 mm.

HARDI data were pre-processed in ExploreDTI v4.8.3 (Leemans et al., 2009). Data were corrected for motion and eddy currents prior to tractography. Motion artefacts and eddy current distortions were corrected with B-matrix rotation (Leemans and Jones, 2009); field inhomogeneities were also corrected using standard approaches(Wu et al., 2008). Diffusion weighted images (DWIs) were non-linearly warped to a synthetic T1-weigted image computed from the quantitative T1 map from the DESPOT processing pipeline (see above) and using the fractional anisotropy map computed from the DWIs as a reference. Warps were computed using ‘Elastix’ (Klein et al., 2010) using normalised mutual information as the cost function and constraining deformations to the phase-encoding direction. The corrected DWIs are therefore in the same (undistorted) space as the mcDESPOT images.

The corrected HARDI data were fitted to the diffusion tensor (DT) and corrected for CSF-partial volume effects (Pasternak et al., 2009) was applied to the DTs. The mean diffusivity (MD) was then computed from the DT. Intra-scan head motion was quantified and assessed for potential impact on subsequent statistics (see Supplementary material, Section 4).

### 3.4 Voxel Based Morphometry (VBM).

Differences in GM were analysed using Voxel Based Morphometry (VBM) (Ashburner and Friston, 2000, Good et al., 2001) using an optimised VBM protocol in FSL (Douaud et al., 2007). T1-weighted structural images were skull stripped (Smith, 2002) and GM segmented (Zhang et al., 2001). The GM images were then non-linearly registered to the MNI152 brain template and a study-specific template was created from the average of the transformed GM images. All native GM images were non-linearly registered to this study-specific template and “modulated” to correct for local expansion and contraction due to non-linear components of the spatial transformation. The modulated GM images were then smoothed with an isotropic Gaussian kernel with a FWHM of 4mm^3^.

MD and R1 maps were also analysed with VBM. The MD and R1 data were registered to the original FSPGR T1-wieghted space using a non-rigid affine transform with the synthetic T1-wighted image as a reference. The grey matter segmentation from the FSPGR was then used to mask the grey matter in each image. The images were then registered to the VBM template using the same warp fields originally used to register the GM images. R1 and MD images were then smoothed with Gaussian kernels of 4mm^3^ and 8mm^3^, respectively, to accommodate differences in voxel size. Unlike the GM volume images, modulation was not performed for these images as these variables are not quantifying spatial properties, unlike GM volume. Modulation is therefore not appropriate for these images.

### 3.5 Statistical analysis

Statistical analyses were performed using a voxel-wise general linear model on the template-space images. Multiple comparisons across voxels were corrected using permutation-based non-parametric testing (Nichols and Holmes, 2002), with threshold-free cluster enhancement (Smith and Nichols, 2009) across 5000 permutations.

Two designs were tested. The first treats PE status as a binary classification (with PEs vs. without PEs). The second model treats the PE status as a 4-point ordinal scale (no PEs > suspected > definite > ‘clinical disorder’). In both cases, age and gender were treated as covariates. Maps of permutation corrected *p*-values (*p*_corr_) were computed for each image metric (MD and R1) and each design (i.e. binary or 4-point ordinal classification). Effects are treated as significant at *p*_corr_ < 0.05. In the cases where was a significant correlation between a risk-factor and PEs, the analysis was also performed with the inclusion of the relevant risk factor as a covariate. In addition, correlations between GM measurements and the other risk factors were tested for, with age and gender treated as covariates.

## 4 Results

### 4.1 Demographics

Group effects on each of the candidate risk factors are reported in Table 1. Premorbid IQ, CIS-R scores, parental social class and maternal education all show strong effects in both the binary and the ordinal classifications (*p* < 0.01). A smaller, but still significant, group effect was also observed for birth weight (*p* < 0.05). All other risk factors showed no significant group effects.

### 4.2 Binary classification

**Fig. 1:**
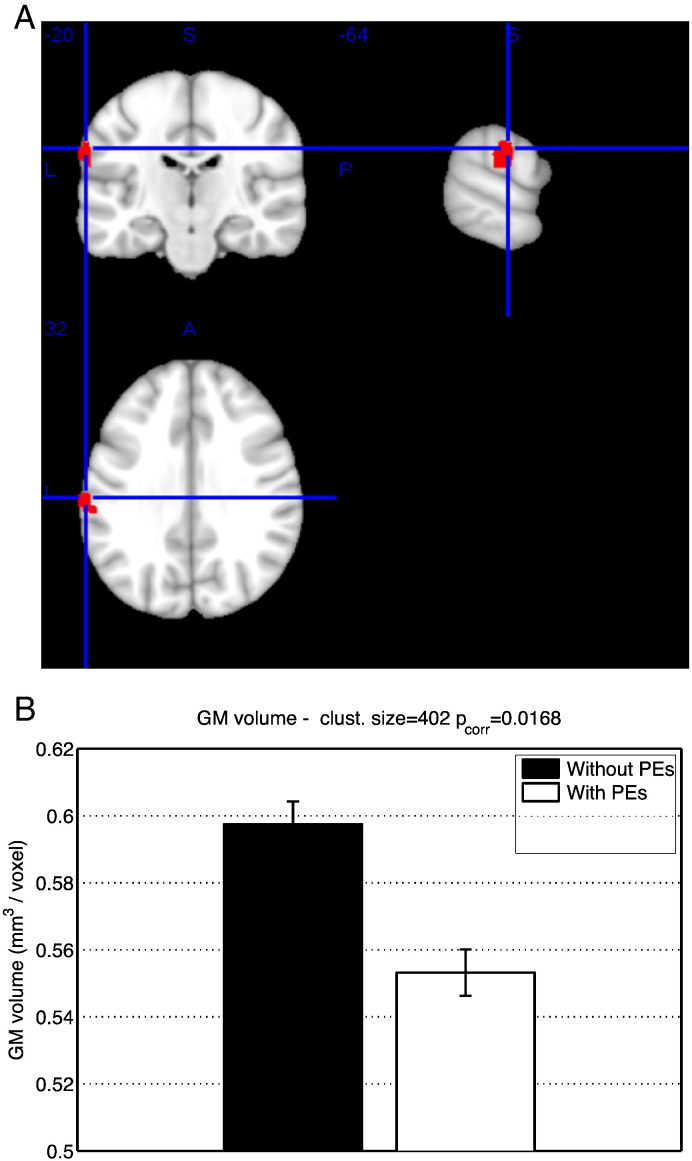
(A) Significant negative effects of PEs on GM volume using the binary classification of PEs. ROI volume 1976 mm^3^. MNI coordinates [− 64 − 20 32]. (B). mean and standard error of GM volume in significant ROI, with binary classification of PEs.

GM volume in a region of the left supramarginal gyrus (Fig. 1, Table 2) was decreased in subjects with PEs compared to subjects with no PEs. There were no group effects on MD or R1.

### 4.3 Ordinal model

**Fig. 2:**
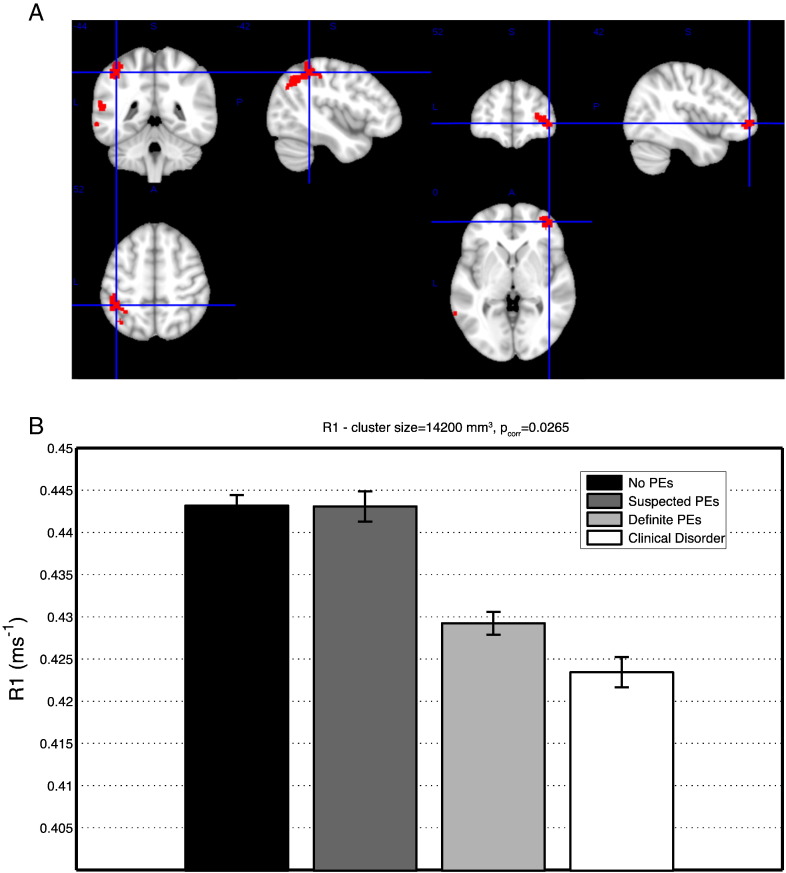
(A) Regions of significant effects of PEs on R1 inferred using the ordinal classification of PEs in left parietal and right frontal cortices. Parietal ROI: volume 11,088 mm^3^, MNI coordinates [− 42 − 44 52], Frontal ROI: volume 2696mm^3^ MNI coordinates [42 52 0]. (B) Mean and standard error of R1 value in the significant ROI, with ordinal classification of PEs.

There was a significant negative effect in R1 in left temporoparietal and right pre-frontal regions (Fig. 2, Table 3), with R1 lowest for the suspected and definite PE groups. No significant effects were identified with GM volume or MD.

### 4.4 Correlation with risk factors

**Fig. 3:**
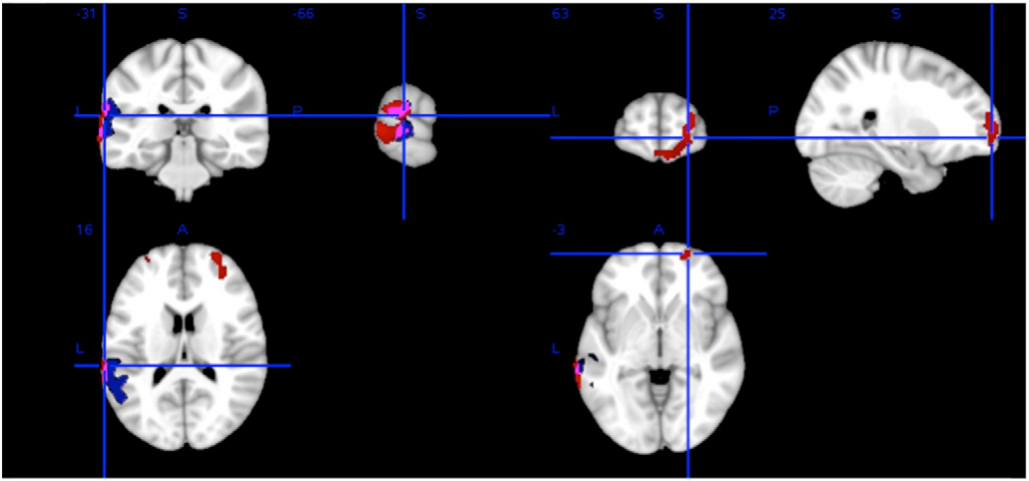
Regions of significant negative correlation between R1 and CIS-R score (red) and significant positive correlation between R1 and maternal education (blue). Overlap between the two Is violet. (For interpretation of the references to color in this figure legend, the reader is referred to the web version of this article.)

There were significant negative correlations between R1 and CIS-R and significant positive correlations between R1 and maternal education. Both these correlations overlap spatially with the correlations with PE status, although in the case of maternal education, the effect is limited to the left temporoparietal region (Fig. 3 and Table 4).

In addition, there were widespread positive correlations between MD and birth-weight, mostly in posterior cingulate, parietal and occipital cortices [data not shown] which did not overlap with the R1 changes nor were they associated with PEs. No other significant correlations were identified.

## 5 Discussion

This study used structural MRI, T1 relaxometry and diffusion tensor imaging (DTI) in conjunction with voxel-based morphometry (VBM) to characterise GM macrostructure and microstructure associated with psychotic experiences (PEs) in a large population-based sample of young adults.

Reduction in GM volume in this otherwise healthy group with PEs, centred on a single region: the left supramarginal gyrus, a key component of the ‘heteromodal association cortex’ regarded by some as being selectively affected in the pathological anatomy of schizophrenia (Buchanan et al., 2004). GM Abnormalities in those at increased genetic risk (Bhojraj et al., 2011), have also been detected in this region. However, when we compared our simple binary- with an ordinal-based model closer to the idea of a continuum of psychopathology, the binary model of PEs was associated with reduced volume whereas the ordinal model was not.

Alternately, it was the ordinal model that showed a reduction in cortical R1 in non-overlapping left temporoparietal and right prefrontal regions also noted to show changes in those at high risk of psychosis using conventional volumetric MRI. Such a broad topography of cortical regions has the potential to impinge on many important linguistic and executive functions. The discrepancy between our two main findings might be interpreted as different neuroanatomical substrates for general psychopathology, and more specific pathology relating to psychosis. However, there were no significant relationships with GM volume and CIS-R, or any other risk factors.

The regions where R1 is reduced with PEs are consistent with some of those previously found to show cortical pathology in psychosis risk-groups (Fusar-Poli et al., 2011, Smieskova et al., 2010), particularly pre-frontal and superior temporal regions. It is possible that GM volume reduction becomes more apparent at later stages of development. A meta-analysis (Fusar-Poli et al., 2011) found that GM volume was significantly reduced in older at-risk groups (25 + years) compared to younger at-risk groups.

The interpretation that reduced R1 is reflective of reductions in cortical myelination (Lutti et al., 2014) is consistent with previous histological and post-mortem studies (see (Bakhshi and Chance, 2015, Esiri and Crow, 2008, Harrison and Harrison, 1999) for reviews). Of relevance are studies that have shown evidence of reduced oligodendrocyte number (Hof et al., 2002, Hof et al., 2003, Uranova et al., 2004), notably in frontal cortex particularly affecting layer VI, but not in the adjacent white-matter (Uranova et al., 2004). This could be a pathological corollary of the R1 relaxometry findings. More evidence exists of reductions in myelin basic protein (MBP) staining in schizophrenia (Chambers and Perrone-Bizzozero, 2004, Honer et al., 1999) and altered expression of myelin-related genes (Hakak et al., 2001, Katsel et al., 2005, Roussos and Haroutunian, 2014).

In addition, there is evidence that the developmental trajectory of myelination predicts the trajectory of grey matter volume (Gilmore et al., 2012, Sowell et al., 2003), although this relationship is complex (Deoni et al., 2015). It is possible that changes seen in cortical myelination will manifest as changes in cortical volume later in life and there is some evidence supporting this in our data (see Supplementary material, Section 5). Changes in cortical myelination may be related to synaptic pruning in late adolescence (Huttenlocher and Dabholkar, 1997, Paus et al., 2008), which leads to later reductions in cortical volume. The effects observed in the present study may reflect either a delay or a reduction of this process. Further imaging of the cohort will help verify this. It is also interesting to note that the temporoparietal region where the effect of R1 is seen, has been identified as having the most protracted rate of cortical development (Sowell et al., 2003) which might render it vulnerable to a range of insults over time. Furthermore the absence of correlations between RI measures and early neurodevelopmental factors (birthweight and premorbid IQ at age 8) indicate that such factors are not implicated in the proposed myelin-related pathogenesis of psychosis which occurs at developmental stages closer to the age of the cohort. It is important to note that that T1 relaxation is also sensitive to other factors such as macromolecular composition (proteins and lipids) (Bottomley et al., 1984, Rooney et al., 2007) and iron content (Gelman et al., 2001), so the interpretation of reduced R1 reflecting reduced cortical myelination should be made with caution.

The correlation analysis revealed significant associations between CIS-R score and maternal education, a proxy for socio-economic status, on R1, in regions overlapping with those where the main effect with PEs was observed. It is therefore possible that myelination, and possibly the neurodevelopmental processes described above, are delayed or diminished by poor socioeconomic environment, perhaps via physiological stress mechanisms, e.g., infection and inflammation (Najjar and Pearlman, 2014). The correlations with these factors and the absence of any cortical R1 effects when covarying for these factors suggest myelination, macromolecular composition or iron content is related to more general psychopathology and socioeconomic influences rather than specifically psychosis. The absence of any apparent effect in cortical diffusivity, suggests that change in cell density is not necessarily associated with PEs when seen in a non-clinical context. As with GM volume change, this may be something that does not manifest until later stages of illness. Again longitudinal studies will help clarify this.

There were negative correlations between cortical diffusivity and birth weight, indicating a possible early neurodevelopment influence on the cytoarchitecture of mature cortex, but we found no relationship with psychopathology, despite such an association being documented (Abel et al., 2010, Rifkin et al., 1994). Furthermore, this effect appeared to be independent of any other measured properties in cortex.

The study has a number of strengths, namely the size and population base of the sample uncontaminated by for example medication exposure. Psychopathology was verified using a structured clinical interview and there was extensive socio-demographic and clinical data available on the sample. The imaging methodology was state-of-the-art and included novel application of measures to at-risk groups for psychosis. Weaknesses include the cross sectional nature of the imaging data and the narrow age-range of the participants limiting the developmental inferences that can be drawn. A further limitation is the lack of follow-up information on the cohort, pertaining to progression or remission of PEs. As a result the clinical significance of PEs is unclear. In particular, the risk of a later UHR state, or psychotic disorder is unknown.

In conclusion, there is evidence of a link between cortical volume and R1 in cortex and verified psychotic experiences in young adults drawn from the general population. The alterations in R1 may be reflective of decreased cortical myelination, although macromolecular composition may also be responsible. The effect is consistent with an altered neurodevelopmental trajectory, prior to measureable changes in cortical volume as typically seen in schizophrenia patients and ultra high risk groups. There is also evidence that this marker of neurodevelopment is affected by socioeconomic environment.

## Financial declarations

All authors report no biomedical financial interests or potential conflicts of interest.

## Figures and Tables

**Table 1:** Descriptive and inferential statistics for the candidate covariates. Descriptive statistics for continuous variables report mean ± standard error for each group. Descriptive statistics for ordinal and categorical variables are reported as tabulated frequencies. Inferential statistics for the 2-group binary classification uses the student *t*-test for continuous, the χ^2^-test for categorical and the Cochrane-Armitage χ^2^-test (denoted χ^2^_CM_) for ordinal variables. Inferential statistics for the 4-group ordinal classification uses Spearman's ρ correlation for continuous and ordinal variables and χ^2^_CM_ test for categorical variables.

Variable	Descriptive statistics					Inferential statistics		Proportion imputed
	No PEs	With PEs				2-Group binary classification	4-Group ordinal classification	
		Suspect	Definite	Clinical	Total			
1. Premorbid IQ	111.44 ± 0.12	104.65 ± 0.31	106.79 ± 0.32	103.13 ± 0.42	105.07 ± 0.12	*t* = 3.456 *p* = 0.001⁎⁎	*ρ* = − 0.188 *p* = 0.003⁎⁎	0.137
2. CIS-R score	6.30 ± 0.05	9.16 ± 0.17	12.26 ± 0.18	16.78 ± 0.34	12.33 ± 0.08	*t* = − 5.903 *p* = 1.2 × 10^− 8⁎⁎^	*ρ* = 0.408 *p* = 2.3 × 10^− 11⁎⁎^	0.065
3. Parental social class								0.173
I	10	4	5	0	9	*χ*^2^_CM_ = 9.229, *p* = 0.002⁎⁎	*ρ* = 0.204, *p* = 0.001⁎⁎	
II	54	10	16	5	31			
III (non-manual)	52	24	23	20	67			
III (manual)	6	0	0	2	2			
IV	3	5	2	3	10			
V	0	1	1	2	4			
4. Highest maternal education								0.065
None	11	8	5	5	18	*χ*^2^_CM_ = 9.022, *p* = 0.003⁎⁎	*ρ* = − 0.197, *p* = 0.002⁎⁎	
Vocational	6	5	3	1	9			
Ordinary Level	39	16	21	19	56			
Advanced Level	34	7	7	4	18			
Degree	35	8	11	3	22			
5. Birth weight (g)	3466.2 ± 4.0	3323.4 ± 12.6	3401.7 ± 9.7	3165.3 ± 18.5	3312.2 ± 4.3	*t* = 2.335, *p* = 0.020⁎	*ρ* = − 0.155, *p* = 0.015⁎	0.060
6. Resuscitated at birth								0.387
No	106	33	38	27	98	*χ*^2^ = 1.538, *p* = 0.463	*χ*^2^_CM_ = 0.141, *p* = 0.707	
Yes	19	11	9	5	25			
7. Stressful life events								0.173
No	111	36	40	23	99	*χ*^2^ = 3.301, *p* = 0.069	*χ*^2^_CM_ = 4.451, *p* = 0.035⁎	
Yes	14	8	7	9	24			
8. Handedness								0.411
Right	92	36	34	22	92	*χ*^2^_CM_ = 0.001, *p* = 0.971	*ρ* = 0.022, *p* = 0.737	
No dominance	25	6	9	7	22			
Left	8	2	4	3	9			
9. aTobacco consumption (cigarettes per day)	0.59 ± 0.02	1.27 ± 0.08	0.44 ± 0.04	2.23 ± 0.14	1.20 ± 0.03	*t* = − 1.717, *p* = 0.087	*ρ* = 0.021, *p* = 0.737	0.472
10. bCannabis consumption								0.411
Never	87	28	32	19	79	*χ*^2^_CM_ = 1.588, *p* = 0.208	*ρ* = 0.077, *p* = 0.226	
Once or twice	11	6	4	1	11			
Less than monthly	25	6	8	10	24			
Monthly or more	2	4	3	2	9			
11. cAlcohol consumption								0.020
Never	16	6	2	0	8	*χ*^2^_CM_ = 1.045, *p* = 0.307	*ρ* = 0.053, *p* = 0.408	
Once or twice	23	5	9	6	20			
Less than monthly	12	5	9	6	20			
Monthly	52	21	19	14	54			
Weekly or more	22	7	8	6	21			
12. dMonth of birth	2.30 ± 7.52	1.72 ± 7.33	1.31 ± 7.50	2.18 ± 7.51	1.71 ± 7.44	*F* = 1.565, *p* = 0.212	*ρ* = 0.088, *p* = 0.384	0

Standard covariates (included in all analyses)								
Age (years)	20.11 ± 0.004	20.14 ± 0.013	19.88 ± 0.011	20.04 ± 0.015	20.01 ± 0.004	*t* = 1.488, *ρ* = 0.138	*ρ* = − 0.147, *p* = 0.020⁎	0
Gender								0
Male	49	14	16	7	37	χ^2^ = 2.2757, p = 0.131	χ^2^_CM_ = 2.952, ρ = 0.085	
Female	76	30	31	25	86			

a The number of cigarettes participant smokes every day on average in the last 30 days.

b From a multiple-choice question asking how many times the participant used cannabis in the past 12 months (all substance use data gathered at age 17/18).

c 6 or more units of alcohol in the past year.

d As a scale from 0 (1st January) to 12 (31st December). A Watson-Williams 2-sample F-test and a circular-linear. *ρ*-correlation using the circular statistics toolbox (Berens and Baclawski, 2009).

⁎ *p* < 0.05.

⁎⁎ *p* < 0.01.

**Table 2:** Regions of AAL atlas overlapping with regions of significant effect on GM volume, using the binary classification of PEs.

Region	Overlap with ROI (# voxels)	min p_crr_	MNI coordinates
Supramarginal gyrus (left)	164	0.027	− 64 -20 32
Postcentral (left)	26	0.031	− 62 -18–30
Superior temporal (left)	20	0.031	− 56 -24–18

**Table 3:** Regions of the AAL atlas where R1 significantly correlates with PEs.

Region	Overlap with ROI (# voxels)	min p_corr_	MNI coordinates
Parietal/temporal			
Inferior parietal (left)	399	0.026	− 42 ·-44 52
Middle temporal (left)	392	0.028	− 62 -50 − 10
Angular gyrus (left)	257	0.031	− 52 -52 − 28
Superior temporal (left)	133	0.028	− 58 -48 − 16
Superior parietal (left)	51	0.032	− 38 -44 − 62
Middle occipital (left)	48	0.038	− 36 -70 − 36
Supramarginal gyrus (left)	46	0.031	− 54 -50 − 26
Postcentral gyrus (left)	45	0.032	− 42 -38 − 58

Frontal			
Middle frontal (right)	187	0.032	40 52 − 2
Medial orbitofrontal (right)	64	0.032	42 52 0
Superior frontal (right)	61	0.042	32 -52 − 10
Inferior frontal operculum (left)	38	0.044	− 46 14 − 18
Inferior orbitofrontal (right)	22	0.034	40 48 0

(See Supplementary material, Section 3).

**Table 4:** Regions of the AAL atlas where R1 significantly correlates with CIS-R and maternal education.

Region	Negative correlation with CIS-R score			Positive correlation with maternal education		
	Overlap with ROI (# voxels)	min p_corr_	MNI coordinates	Overlap with ROI (# voxels)	min p_corr_	MNI coordinates
Frontal						
Superior frontal (right)	262	0.024	24 64 − 2	0	–	–
Superior orbitofrontal (right)	170	0.026	24 64 0	0	–	–
Middle frontal (left)	30	0.046	− 28 56 − 14	0	–	–
Middle frontal (right)	216	0.024	32 58 − 20	0	–	–
Middle orbitofrontal (right)	84	0.032	10 64 14	0	–	–
Inferior orbitofrontal (right)	11	0.048	48 50 10	0	–	–
Medial orbitofrontal (right)	34	0.030	10 64 14	0	–	–
Gyrus rectus (left)	26	0.030	2 64 16	0	–	–
Gyrus rectus (right)	30	0.028	4 66 16	0	–	–

Parietal/temporal						
Supramarginal gyrus (left)	77	0.020	-64 -34 − 24	216	0.022	− 54 -38 − 28
Superior temporal (left)	105	0.018	− 64 -40 − 20	204	0.026	− 60 -36 − 18
Middle temporal (left)	352	0.016	− 68 -38 − 4	176	0.034	− 52 -56 − 20

## Appendix A Supplementary data

Supplementary material. Image 1

## References

[bb0005] Abel K.M., Wicks S., Susser E.S., Dalman C., Pedersen M.G., Mortensen P.B., Webb R.T. (2010). Birth weight, schizophrenia, and adult mental disorder: is risk confined to the smallest babies?. Arch. Gen. Psychiatry.

[bb0010] Ashburner J., Friston K.J. (2000). Voxel-based morphometry—the methods. NeuroImage.

[bb0015] Bakhshi K., Chance S.A. (2015). The neuropathology of schizophrenia: a selective review of past studies and emerging themes in brain structure and cytoarchitecture. Neuroscience.

[bb0020] Basser P.J., Mattiello J., LeBihan D. (1994). MR diffusion tensor spectroscopy and imaging. Biophys. J..

[bb0025] Berens P., Baclawski K. (2009). CircStat: a MATLAB toolbox for circular statistics. J. Stat. Softw..

[bb0030] Bhojraj T.S., Sweeney J.A., Prasad K.M., Eack S., Rajarethinam R., Francis A.N., Montrose D.M., Keshavan M.S. (2011). Progressive alterations of the auditory association areas in young non-psychotic offspring of schizophrenia patients. J. Psychiatr. Res..

[bb0035] Borgwardt S.J., McGuire P.K., Aston J., Berger G., Dazzan P., Gschwandtner U., Pflüger M., D'Souza M., Radue E.-W., Riecher-Rössler A. (2007). Structural brain abnormalities in individuals with an at-risk mental state who later develop psychosis. Br. J. Psychiatry Suppl..

[bb0040] Borgwardt S., McGuire P., Fusar-Poli P. (2011). Gray matters!—mapping the transition to psychosis. Schizophr. Res..

[bb0045] Bottomley P.A., Foster T.H., Argersinger R.E., Pfeifer L.M. (1984). A review of normal tissue hydrogen NMR relaxation times and relaxation mechanisms from 1–100 MHz: dependence on tissue type, NMR frequency, temperature, species, excision, and age. Med. Phys..

[bb0050] Boyd A., Golding J., Macleod J., Lawlor D.a., Fraser A., Henderson J., Molloy L., Ness A., Ring S., Smith G.D. (2013). Cohort profile: the “children of the 90s”-the index offspring of the Avon longitudinal study of parents and children. Int. J. Epidemiol..

[bb0055] Buchanan R.W., Francis A., Arango C., Miller K., Lefkowitz D.M., McMahon R.P., Barta P.E., Pearlson G.D. (2004). Morphometric assessment of the heteromodal association cortex in schizophrenia. Am. J. Psychiatry.

[bb0060] Buck S.F. (1960). A method of estimation of missing values in multivariate data suitable for use with an electronic computer. J. R. Stat. Soc. Ser. B.

[bb0065] Byrne M., Agerbo E., Eaton W.W., Mortensen P.B. (2004). Parental socio-economic status and risk of first admission with schizophrenia - a Danish national register based study. Soc. Psychiatry Psychiatr. Epidemiol..

[bb0070] Cannon T.D., Chung Y., He G., Sun D., Jacobson A., van Erp T.G.M., McEwen S., Addington J., Bearden C.E., Cadenhead K., Cornblatt B., Mathalon D.H., McGlashan T., Perkins D., Jeffries C., Seidman L.J., Tsuang M., Walker E., Woods S.W., Heinssen R. (2014). Progressive reduction in cortical thickness as psychosis develops: a multisite longitudinal neuroimaging study of youth at elevated clinical risk. Biol. Psychiatry.

[bb0075] Cannon T.D., Chung Y., He G., Sun D., Jacobson A., Van Erp T.G.M., McEwen S., Addington J., Bearden C.E., Cadenhead K., Cornblatt B., Mathalon D.H., McGlashan T., Perkins D., Jeffries C., Seidman L.J., Tsuang M., Walker E., Woods S.W., Heinssen R. (2015). Progressive reduction in cortical thickness as psychosis develops: a multisite longitudinal neuroimaging study of youth at elevated clinical risk. Biol. Psychiatry.

[bb0080] Chambers J.S., Perrone-Bizzozero N.I. (2004). Altered myelination of the hippocampal formation in subjects with schizophrenia and bipolar disorder. Neurochem. Res..

[bb0085] Cropley V.L., Lin A., Nelson B., Reniers R.L.E.P., Yung A.R., Bartholomeusz C.F., Klauser P., Velakoulis D., McGorry P., Wood S.J., Pantelis C. (2016). Baseline grey matter volume of non-transitioned “ultra high risk” for psychosis individuals with and without attenuated psychotic symptoms at long-term follow-up. Schizophr. Res..

[bb0090] Dalman C., Thomas H.V., David A.S., Gentz J., Lewis G., Allebeck P. (2001). Signs of asphyxia at birth and risk of schizophrenia. Population-based case-control study. Br. J. Psychiatry.

[bb0095] David A.S., Malmberg A., Brandt L., Alleback P., Lewis G. (1997). IQ and risk for schizophrenia: a population-based cohort study. Psychol. Med..

[bb0100] Deoni S.C.L. (2007). High-resolution T1 mapping of the brain at 3T with driven equilibrium single pulse observation of T1 with high-speed incorporation of RF field inhomogeneities (DESPOT1-HIFI). J. Magn. Reson. Imaging.

[bb0105] Deoni S.C.L., Rutt B.K., Arun T., Pierpaoli C., Jones D.K. (2008). Gleaning multicomponent T1 and T2 information from steady-state imaging data. Magn. Reson. Med..

[bb0110] Deoni S.C.L., Dean D.C., Remer J., Dirks H., O'Muircheartaigh J. (2015). Cortical maturation and myelination in healthy toddlers and young children. NeuroImage.

[bb0115] Dinse J., Härtwich N., Waehnert M.D., Tardif C.L., Schäfer A., Geyer S., Preim B., Turner R., Bazin P.-L. (2015). A cytoarchitecture-driven myelin model reveals area-specific signatures in human primary and secondary areas using ultra-high resolution in-vivo brain MRI. NeuroImage.

[bb0120] Douaud G., Smith S., Jenkinson M., Behrens T., Johansen-Berg H., Vickers J., James S., Voets N., Watkins K., Matthews P.M., James A. (2007). Anatomically related grey and white matter abnormalities in adolescent-onset schizophrenia. Brain.

[bb0125] Drakesmith M., Caeyenberghs K., Dutt A., Zammit S., Evans C.J., Reichenberg A., Lewis G., David A.S., Jones D.K. (2015). Schizophrenia-like topological changes in the structural connectome of individuals with subclinical psychotic experiences. Hum. Brain Mapp..

[bb0130] Drakesmith M., Dutt A., Fonville L., Zammit S., Reichenberg A., Evans C.J., Lewis G., Jones D.K., David A.S. (2016). Mediation of developmental risk factors for psychosis by white matter microstructure in young adults with psychotic experiences. JAMA Psychiatry.

[bb0135] Ellison-Wright I., Glahn D.C., Laird A.R., Thelen S.M., Bullmore E. (2008). The anatomy of first-episode and chronic schizophrenia: an anatomical likelihood estimation meta-analysis. Am. J. Psychiatry.

[bb0140] Esiri M., Crow T., Love S., Louis D., Ellison D.W. (2008). Psychiatric disease.

[bb0145] Foerster A., Lewis S.W., Owen M.J., Murray R.M. (1991). Low birth weight and a family history of schizophrenia predict poor premorbid functioning in psychosis. Schizophr. Res..

[bb0150] Fusar-Poli P., Borgwardt S., Crescini A., Deste G., Kempton M.J., Lawrie S., McGuire P., Sacchetti E. (2011). Neuroanatomy of vulnerability to psychosis: a voxel-based meta-analysis. Neurosci. Biobehav. Rev..

[bb0155] Fusar-Poli P., Borgwardt S., Bechdolf A., Addington J., Riecher-Rössler A., Schultze-Lutter F., Keshavan M., Wood S., Ruhrmann S., Seidman L.J., Valmaggia L., Cannon T., Velthorst E., De Haan L., Cornblatt B., Bonoldi I., Birchwood M., McGlashan T., Carpenter W., McGorry P., Klosterkötter J., McGuire P., Yung A. (2013). The psychosis high-risk state: a comprehensive state-of-the-art review. JAMA psychiatry.

[bb0160] Gallagher B.J., Jones B.J., Pardes M. (2013). Stressful life events, social class and symptoms of schizophrenia. Clin. Schizophr. Relat. Psychoses.

[bb0165] Gelman N., Ewing J.R., Gorell J.M., Spickler E.M., Solomon E.G. (2001). Interregional variation of longitudinal relaxation rates in human brain at 3.0 T: relation to estimated iron and water contents. Magn. Reson. Med..

[bb0170] Gilmore J.H., Shi F., Woolson S.L., Knickmeyer R.C., Short S.J., Lin W., Zhu H., Hamer R.M., Styner M., Shen D. (2012). Longitudinal development of cortical and subcortical gray matter from birth to 2 years. Cereb. Cortex.

[bb0175] Golding J., Pembrey M., Jonesa R., The ALSPAC Study Team (2001). ALSPAC-the Avon longitudinal study of parents and children. Paediatr. Perinat. Epidemiol..

[bb0180] Golombok S., Rust J., Wechsler D. (1992). The UK revision. The Manual of the Wechsler Intelligence Scale for Children - 3rd Revision.

[bb0185] Good C.D., Johnsrude I.S., Ashburner J., Henson R.N., Friston K.J., Frackowiak R.S. (2001). A voxel-based morphometric study of ageing in 465 normal adult human brains. NeuroImage.

[bb0190] Goodman R., Ford T., Richards H., Gatward R., Meltzer H. (2000). The development and well-being assessment: description and initial validation of an integrated assessment of child and adolescent psychopathology. J. Child Psychol. Psychiatry.

[bb0195] Hakak Y., Walker J.R., Li C., Wong W.H., Davis K.L., Buxbaum J.D., Haroutunian V., Fienberg A.A. (2001). Genome-wide expression analysis reveals dysregulation of myelination-related genes in chronic schizophrenia. Proc. Natl. Acad. Sci..

[bb0200] Harrison P.J., Harrison P.J. (1999). The neuropathology of schizophrenia: a critical review of the data and their interpretation. Brain.

[bb0205] Hof P.R., Haroutunian V., Copland C., Davis K.L., Buxbaum J.D. (2002). Molecular and cellular evidence for an oligodendrocyte abnormality in schizophrenia. Neurochem. Res..

[bb0210] Hof P.R., Haroutunian V., Friedrich V.L., Byne W., Buitron C., Perl D.P., Davis K.L. (2003). Loss and altered spatial distribution of oligodendrocytes in the superior frontal gyrus in schizophrenia. Biol. Psychiatry.

[bb0215] Homer J., Beevers M.S. (1985). Driven-equilibrium single-pulse observation of T1 relaxation. A reevaluation of a rapid “new” method for determining NMR spin-lattice relaxation times. J. Magn. Reson..

[bb0220] Honea R., Crow T.J., Passingham D., Mackay C.E. (2005). Regional deficits in brain volume in schizophrenia: a meta-analysis of voxel-based morphometry studies. Am. J. Psychiatry.

[bb0225] Honer W.G., Falkai P., Chen C., Arango V., Mann J.J., Dwork A.J. (1999). Synaptic and plasticity-associated proteins in anterior frontal cortex in severe mental illness. Neuroscience.

[bb0230] Horwood J., Salvi G., Thomas K., Duffy L., Gunnell D., Hollis C., Lewis G., Menezes P., Thompson A., Wolke D., Zammit S., Harrison G. (2008). IQ and non-clinical psychotic symptoms in 12-year-olds: results from the ALSPAC birth cohort. Br. J. Psychiatry.

[bb0235] Huttenlocher P.R., Dabholkar A.S. (1997). Regional differences in synaptogenesis in human cerebral cortex. J. Comp. Neurol..

[bb0240] Jenkinson M., Beckmann C.F., Behrens T.E.J., Woolrich M.W., Smith S.M. (2012). FSL. NeuroImage.

[bb0245] Jones D.K., Simmons A., Williams S.C.R., Horsfield M.A. (1999). Non-invasive assessment of axonal fiber connectivity in the human brain via diffusion tensor MRI. Magn. Reson. Med..

[bb0250] Kanaan R.A.A., Kim J.-S., Kaufmann W.E., Pearlson G.D., Barker G.J., McGuire P.K. (2005). Diffusion tensor imaging in schizophrenia. Biol. Psychiatry.

[bb0255] Kang X., Herron T.J., Turken A.U., Woods D.L. (2012). Diffusion properties of cortical and pericortical tissue: regional variations, reliability and methodological issues. Magn. Reson. Imaging.

[bb0260] Katsel P., Davis K.L., Haroutunian V. (2005). Variations in myelin and oligodendrocyte-related gene expression across multiple brain regions in schizophrenia: a gene ontology study. Schizophr. Res..

[bb0265] Kelly C. (2000). Cigarette smoking and schizophrenia. Adv. Psychiatr. Treat..

[bb0270] Klein S., Staring M., Murphy K., Viergever M.A., Pluim J.P.W. (2010). Elastix: a toolbox for intensity-based medical image registration. IEEE Trans. Med. Imaging.

[bb0275] Koskinen J., Löhönen J., Koponen H., Isohanni M., Miettunen J. (2009). Prevalence of alcohol use disorders in schizophrenia—a systematic review and meta-analysis. Acta Psychiatr. Scand..

[bb0280] Kraan T., Velthorst E., Smit F., de Haan L., van der Gaag M. (2015). Trauma and recent life events in individuals at ultra high risk for psychosis: review and meta-analysis. Schizophr. Res..

[bb0285] Leemans A., Jones D.K. (2009). The B-matrix must be rotated when correcting for subject motion in DTI data. Magn. Reson. Med..

[bb0290] Leemans A., Jeurissen B., Sijbers J., Jones D.K. (2009). ExploreDTI: a graphical toolbox for processing, analyzing, and visualizing diffusion MR data.

[bb0295] Lewis G. (2002). Asphyxia at birth and schizophrenia. Br. J. Psychiatry.

[bb0300] Lewis G., Pelosi A.J., Araya R., Dunn G. (1992). Measuring psychiatric disorder in the community: a standardized assessment for use by lay interviewers. Psychol. Med..

[bb0305] Lutti A., Dick F., Sereno M.I., Weiskopf N. (2013). Using high-resolution quantitative mapping of R1 as an index of cortical myelination. NeuroImage.

[bb0310] Lutti A., Dick F., Sereno M.I., Weiskopf N. (2014). Using high-resolution quantitative mapping of R1 as an index of cortical myelination. NeuroImage.

[bb0315] Mechelli A., Riecher-Rössler A., Meisenzahl E.M., Tognin S., Wood S.J., Borgwardt S.J., Koutsouleris N., Yung A.R., Stone J.M., Phillips L.J., McGorry P.D., Valli I., Velakoulis D., Woolley J., Pantelis C., McGuire P. (2011). Neuroanatomical abnormalities that predate the onset of psychosis: a multicenter study. Arch. Gen. Psychiatry.

[bb0320] Meisenzahl E.M., Koutsouleris N., Bottlender R., Scheuerecker J., Jäger M., Teipel S.J., Holzinger S., Frodl T., Preuss U., Schmitt G., Burgermeister B., Reiser M., Born C., Möller H.-J. (2008). Structural brain alterations at different stages of schizophrenia: a voxel-based morphometric study. Schizophr. Res..

[bb0325] Moore T.H.M., Zammit S., Lingford-Hughes A., Barnes T.R.E., Jones P.B., Burke M., Lewis G. (2007). Cannabis use and risk of psychotic or affective mental health outcomes: a systematic review. Lancet.

[bb0330] Morgan C., Fisher H. (2007). Environment and schizophrenia: environmental factors in schizophrenia: childhood trauma—a critical review. Schizophr. Bull..

[bb0335] Muñoz Maniega S., Bastin M.E., Armitage P.A., Farrall A.J., Carpenter T.K., Hand P.J., Cvoro V., Rivers C.S., Wardlaw J.M. (2004). Temporal evolution of water diffusion parameters is different in grey and white matter in human ischaemic stroke. J. Neurol. Neurosurg. Psychiatry.

[bb0340] Najjar S., Pearlman D.M. (2014). Neuroinflammation and white matter pathology in schizophrenia: systematic review. Schizophr. Res..

[bb0345] Nichols T.E., Holmes A.P. (2002). Nonparametric permutation tests for functional neuroimaging: a primer with examples. Hum. Brain Mapp..

[bb0350] Norman R.M., Malla A.K. (1993). Stressful life events and schizophrenia. I: A review of the research. Br. J. Psychiatry.

[bb0355] O'Donoghue B., Fanning F., Lyne J., Renwick L., Madigan K., Kinsella A., Lane A., Turner N., O'Callaghan E., Clarke M. (2015). Social Class at Birth and Risk of Psychosis.

[bb9000] Office of Population Censuses and Surveys (1991). Social Survey Division. (1996).. OPCS Omnibus Survey, September 1991.

[bb0360] Oreja-Guevara C., Rovaris M., Iannucci G., Valsasina P., Caputo D., Cavarretta R., Sormani M.P., Ferrante P., Comi G., Filippi M. (2005). Progressive gray matter damage in patients with relapsing–remitting multiple sclerosis: a longitudinal diffusion tensor magnetic resonance imaging study. Arch. Neurol..

[bb0365] Pantelis C., Velakoulis D., McGorry P.D., Wood S.J., Suckling J., Phillips L.J., Yung A.R., Bullmore E.T., Brewer W., Soulsby B., Desmond P., McGuire P.K. (2003). Neuroanatomical abnormalities before and after onset of psychosis: a cross-sectional and longitudinal MRI comparison. Lancet (London, England).

[bb0370] Park J.Y., Park H.-J., Kim D.-J., Kim J.-J. (2014). Positive symptoms and water diffusivity of the prefrontal and temporal cortices in schizophrenia patients: a pilot study. Psychiatry Res. Neuroimaging.

[bb0375] Pasternak O., Sochen N., Gur Y., Intrator N., Assaf Y. (2009). Free water elimination and mapping from diffusion MRI. Magn. Reson. Med..

[bb0380] Paus T., Keshavan M., Giedd J.N. (2008). Why do many psychiatric disorders emerge during adolescence?. Nat. Rev. Neurosci..

[bb0385] Rifkin L., Lewis S., Jones P., Toone B., Murray R. (1994). Low birth weight and schizophrenia. Br. J. Psychiatry.

[bb0390] Rooney W.D., Johnson G., Li X., Cohen E.R., Kim S.-G., Ugurbil K., Springer C.S. (2007). Magnetic field and tissue dependencies of human brain longitudinal1H2O relaxation in vivo. Magn. Reson. Med..

[bb0395] Roussos P., Haroutunian V. (2014). Schizophrenia: susceptibility genes and oligodendroglial and myelin related abnormalities. Front. Cell. Neurosci..

[bb0400] Rovaris M., Judica E., Gallo A., Benedetti B., Sormani M.P., Caputo D., Ghezzi A., Montanari E., Bertolotto A., Mancardi G., Bergamaschi R., Martinelli V., Comi G., Filippi M. (2006). Grey matter damage predicts the evolution of primary progressive multiple sclerosis at 5 years. Brain.

[bb0405] Satterthwaite T.D., Wolf D.H., Calkins M.E., Vandekar S.N., Erus G., Ruparel K., Roalf D.R., Linn K.A., Elliott M.A., Moore T.M., Hakonarson H., Shinohara R.T., Davatzikos C., Gur R.C., Gur R.E. (2016). Structural brain abnormalities in youth with psychosis spectrum symptoms. JAMA Psychiatry.

[bb0410] Simon A.E., Velthorst E., Nieman D.H., Linszen D., Umbricht D., de Haan L. (2011). Ultra high-risk state for psychosis and non-transition: a systematic review. Schizophr. Res..

[bb0415] Smieskova R., Fusar-Poli P., Allen P., Bendfeldt K., Stieglitz R.D., Drewe J., Radue E.W., McGuire P.K., Riecher-Rössler A., Borgwardt S.J. (2010). Neuroimaging predictors of transition to psychosis—a systematic review and meta-analysis. Neurosci. Biobehav. Rev..

[bb0420] Smith S.M. (2002). Fast robust automated brain extraction. Hum. Brain Mapp..

[bb0425] Smith S.M., Nichols T.E. (2009). Threshold-free cluster enhancement: addressing problems of smoothing, threshold dependence and localisation in cluster inference. NeuroImage.

[bb0430] Sowell E.R., Peterson B.S., Thompson P.M., Welcome S.E., Henkenius A.L., Toga A.W. (2003). Mapping cortical change across the human life span. Nat. Neurosci..

[bb0440] Uranova N.a., Vostrikov V.M., Orlovskaya D.D., Rachmanova V.I. (2004). Oligodendroglial density in the prefrontal cortex in schizophrenia and mood disorders: a study from the Stanley neuropathology consortium. Schizophr. Res..

[bb0445] Haijma S.V., Van Haren N., Cahn W., Koolschijn P.C.M.P., Hulshoff Pol H.E., Kahn R.S. (2013). Brain volumes in schizophrenia: a meta-analysis in over 18 000 subjects. Schizophr. Bull..

[bb0450] Velakoulis D., Wood S.J., Wong M.T.H., McGorry P.D., Yung A., Phillips L., Smith D., Brewer W., Proffitt T., Desmond P., Pantelis C. (2006). Hippocampal and amygdala volumes according to psychosis stage and diagnosis. Arch. Gen. Psychiatry.

[bb0455] Vita A., De Peri L., Silenzi C., Dieci M. (2006). Brain morphology in first-episode schizophrenia: a meta-analysis of quantitative magnetic resonance imaging studies. Schizophr. Res..

[bb0460] Vita A., De Peri L., Deste G., Sacchetti E. (2012). Progressive loss of cortical gray matter in schizophrenia: a meta-analysis and meta-regression of longitudinal MRI studies. Transl. Psychiatry.

[bb0465] Vite C.H., Magnitsky S., Aleman D., O'Donnell P., Cullen K., Ding W., Pickup S., Wolfe J.H., Poptani H. (2008). Apparent diffusion coefficient reveals gray and white matter disease, and T2 mapping detects white matter disease in the brain in feline alpha-mannosidosis. AJNR Am. J. Neuroradiol..

[bb0470] Weiskopf N., Mohammadi S., Lutti A., Callaghan M.F. (2015). Advances in MRI-based computational neuroanatomy. Curr. Opin. Neurol..

[bb0475] Werner S., Malaspina D., Rabinowitz J. (2007). Socioeconomic status at birth is associated with risk of schizophrenia: population-based multilevel study. Schizophr. Bull..

[bb0480] Wing J.K., Babor T., Brugha T., Burke J., Cooper J.E., Giel R., Jablenski A., Regier D., Sartorius N. (1990). SCAN. Schedules for clinical assessment in neuropsychiatry. Arch. Gen. Psychiatry.

[bb0485] Woodberry K.A., Giuliano A.J., Seidman L.J. (2008). Premorbid IQ in schizophrenia: a meta-analytic review. Am. J. Psychiatry.

[bb0490] Wright I.C., Rabe-Hesketh S., Woodruff P.W., David A.S., Murray R.M., Bullmore E.T. (2000). Meta-analysis of regional brain volumes in schizophrenia. Am. J. Psychiatry.

[bb0495] Wu M., Chang L.C., Walker L., Lemaitre H., Barnett A.S., Marenco S., Pierpaoli C. (2008). Comparison of EPI distortion correction methods in diffusion tensor MRI using a novel framework. Med. Image Comput. Comput. Interv..

[bb0500] Zammit S., Allebeck P., David A.S., Dalman C., Hemmingsson T., Lundberg I., Lewis G. (2004). A longitudinal study of premorbid IQ score and risk of developing schizophrenia, bipolar disorder, severe depression, and other nonaffective psychoses. Arch. Gen. Psychiatry.

[bb0505] Zammit S., Horwood J., Thompson A., Thomas K., Menezes P., Gunnell D., Hollis C., Wolke D., Lewis G., Harrison G. (2008). Investigating if psychosis-like symptoms (PLIKS) are associated with family history of schizophrenia or paternal age in the ALSPAC birth cohort. Schizophr. Res..

[bb0510] Zammit S., Kounali D., Cannon M., David A.S., Gunnell D., Heron J., Jones P.B., Lewis S., Sullivan S., Wolke D., Lewis G. (2013). Psychotic experiences and psychotic disorders at age 18 in relation to psychotic experiences at age 12 in a longitudinal population-based cohort study. Am. J. Psychiatry.

[bb0515] Zhang Y., Brady M., Smith S. (2001). Segmentation of brain MR images through a hidden Markov random field model and the expectation-maximization algorithm. IEEE Trans. Med. Imaging.

[bb0520] Ziermans T.B., Durston S., Sprong M., Nederveen H., van Haren N.E.M., Schnack H.G., Lahuis B.E., Schothorst P.F., van Engeland H. (2009). No evidence for structural brain changes in young adolescents at ultra high risk for psychosis. Schizophr. Res..

[bb0525] Ziermans T.B., Schothorst P.F., Schnack H.G., Koolschijn P.C.M.P., Kahn R.S., Van Engeland H., Durston S. (2012). Progressive structural brain changes during development of psychosis. Schizophr. Bull..

