# Supplementary material for: Volumetric, relaxometric and diffusometric correlates of psychotic experiences in a non-clinical sample of young adults
Source: Neuroimage Clin. 2016 Sep 4;12:550–8. doi: 10.1016/j.nicl.2016.09.002 (PMC5031471; doi:10.1016/j.nicl.2016.09.002)
Supplement: Supplementary file 1 — Supplementary material. [file mmc1.docx]

# Supplementary material

# Drakesmith et al, “Volumetric, relaxometric and diffusometric correlates of psychotic experiences in a non-clinical sample of young adults.”

# NeuroImage Clinical.

Table of Contents

1. Description of ALSPAC cohort 1

2. Description of full mcDESPOT protocol 3

3. Significant effect with inclusion of risk factors as covariates 4

4. Data quality across PE status 4

5. GM and R1 data at respective site of peak effects 6

## 1. Description of ALSPAC cohort

ALSPAC recruited 14,541 pregnant women resident in Avon, UK with expected dates of delivery 1st April 1991 to 31st December 1992. 14,541 is the *initial* number of pregnancies for which the mother enrolled in the ALSPAC study and had either returned at least one questionnaire or attended a “Children in Focus (CiF)” clinic by 19/07/99. Of these *initial* pregnancies, there were a total of 14,676 foetuses, resulting in 14,062 live births and 13,988 children who were alive at 1 year of age.

When the oldest children were approximately 7 years of age, an attempt was made to bolster the initial sample with eligible cases who had failed to join the study originally. As a result, when considering variables collected from the age of seven onwards (and potentially abstracted from obstetric notes) there are data available for more than the 14,541 pregnancies mentioned above.

The number of new pregnancies not in the initial sample (known as Phase I enrolment) that are currently represented on the built files and reflecting enrolment status at the age of 18 is 706 (452 and 254 recruited during Phases II and III respectively), resulting in an additional 713 children being enrolled. The phases of enrolment are described in more detail in the cohort profile paper(Boyd et al., 2013).

The total sample size for analyses using any data collected after the age of seven is therefore 15,247 pregnancies, resulting in 15,458 foetuses. Of this total sample of 15,458 foetuses, 14,775 were live births and 14,701 were alive at 1 year of age.

A 10% sample of the ALSPAC cohort, the CiF group, attended clinics at the University of Bristol at various time intervals between 4 to 61 months of age. The CiF group were chosen at random from the last 6 months of ALSPAC births (1432 families attended at least one clinic). Excluded were those mothers who had moved out of the area or were lost to follow-up, and those participating in another study of infant development in Avon.

References

Boyd, A., Golding, J., Macleod, J., Lawlor, D. a., Fraser, A., Henderson, J., Molloy, L., Ness, A., Ring, S., Smith, G.D., 2013. Cohort profile: The “Children of the 90s”-The index offspring of the avon longitudinal study of parents and children. Int. J. Epidemiol. 42, 111–127.

## 2. Description of full mcDESPOT protocol

The DESPOT1 protocol described in the main text constitutes part of the mcDESPOT protocol. This was used to derived myelin water fraction (MWF) maps. MWF maps are an alternative method for imaging myelin. In the present study, MWF were analysed in the same way as R1.

Multi-component driven equilibrium single pulse observation of T_1_ and T_2_ (mcDESPOT) (Deoni et al., 2008) was acquired with Spoiled Gradient Recall (SPGR) images across eight flip angles, one inversion recovery SPGR (IR-SPGR) and steady-state free precession (SSFP) images across eight flip angles and two phase-cycling angles were acquired. A total of 25 images were acquired for each subject. All images were acquired in sagittal orientation with a slice matrix of 128x128 (1.72x1.72mm resolution) with a minimum of 88 slices (slice thickness = 1.7mm). Additional slices were added for some subjects to ensure full head coverage.

Sequence-specific parameters were: SPGR: TE=2.112ms, TR=4.7ms, flip angles = 3°, 4°, 5°, 6°, 7°, 9°, 13° and 18°. IR-SPGR: TE=2.112ms, TR=4.7ms, IR=450ms, flip angle = 5°. SSFP: TE = 1.6ms TR=3.2ms, flip angles of 10.59°, 14.12°, 18.53°, 23.82° 29.12° 35.29°, 45°, 60° and phase-cycling angles of 0° and 180°.

All images were linearly co-registered to the SPGR image acquired with a flip-angle of 13° to correct for subject motion. Non-brain tissue was removed using a mask computed with the BET algorithm(Smith, 2002). Registration and brain masking were performed with FSL (<http://www.fmrib.ox.ac.uk/fsl/>). Images were then corrected for B1 inhomogeneities and off-resonance artefacts, using maps generated from the IR-SPGR and 2 phase-cycling SSFP acquisitions, respectively. The mcDESPOT algorithm(Deoni et al., 2008) was then used to identify the fast (water trapped within the myelin layers) and slow (free-moving water in intra- and extra-cellular space) components of the T1 and T2 decay and the corresponding volume fractions. The fast volume fraction was taken as a map of the myelin water fraction (MWF).

No significant effects were observed in MWF, in contrast to the R1 findings. We suggest this is due differences in the statistical properties of the MWF and R1. MWF has lower statistical power than R1 and the required sample size to observe a particular effect size in MWF is considerably larger than for R1. Are more detailed account of this issue is presented in (De Santis et al., 2014) Other researcher have pointed out that that estimates of quantities T2 from SSFP may be biased and therefore yield inaccurate MWF estimates (Zhang et al., 2015) and that the precision of MWF estimates lack precision due to the larger number of parameters being fitted (Lankford and Does, 2013).

References

De Santis, S., Drakesmith, M., Bells, S., Assaf, Y., Jones, D.K., 2014. Why diffusion tensor MRI does well only some of the time: Variance and covariance of white matter tissue microstructure attributes in the living human brain. Neuroimage 89, 35–44.

Deoni, S.C.L., Rutt, B.K., Arun, T., Pierpaoli, C., Jones, D.K., 2008. Gleaning multicomponent T1 and T2 information from steady-state imaging data. Magn. Reson. Med. 60, 1372–87.

Lankford, C.L., Does, M.D., 2013. On the inherent precision of mcDESPOT. Magn. Reson. Med. 69, 127–36.

Smith, S.M., 2002. Fast robust automated brain extraction. Hum. Brain Mapp. 17, 143–55.

Zhang, J., Kolind, S.H., Laule, C., MacKay, A.L., 2015. How does magnetization transfer influence mcDESPOT results? Magn. Reson. Med. 74, 1327–1335.

## 3. Significant effect with inclusion of risk factors as covariates

Figure S1 shows regional effect of PEs equivalent to figure 2 in the main text, but with covariance for risk factors which were also found to be correlated with PEs. This was done to ensure that the effects of PEs reported in the main text are not simply due to the effects of these other risk factors. Significant effects were still found in the same regions when covarying for premorbid IQ and birthweight but not for CIS-R score, maternal education or parental social class.


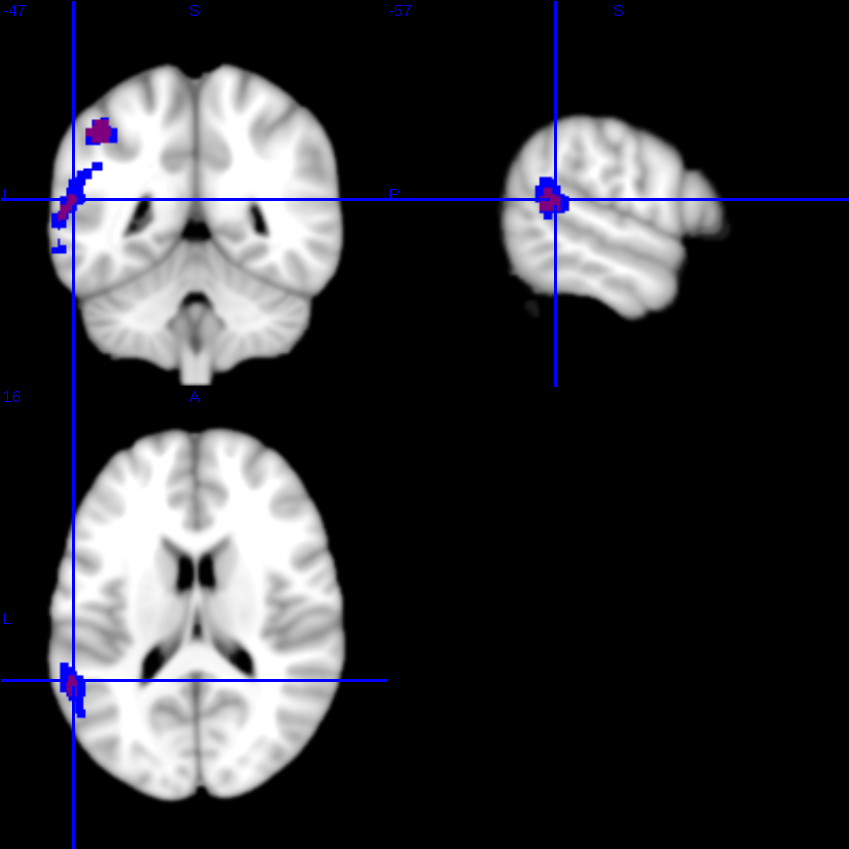

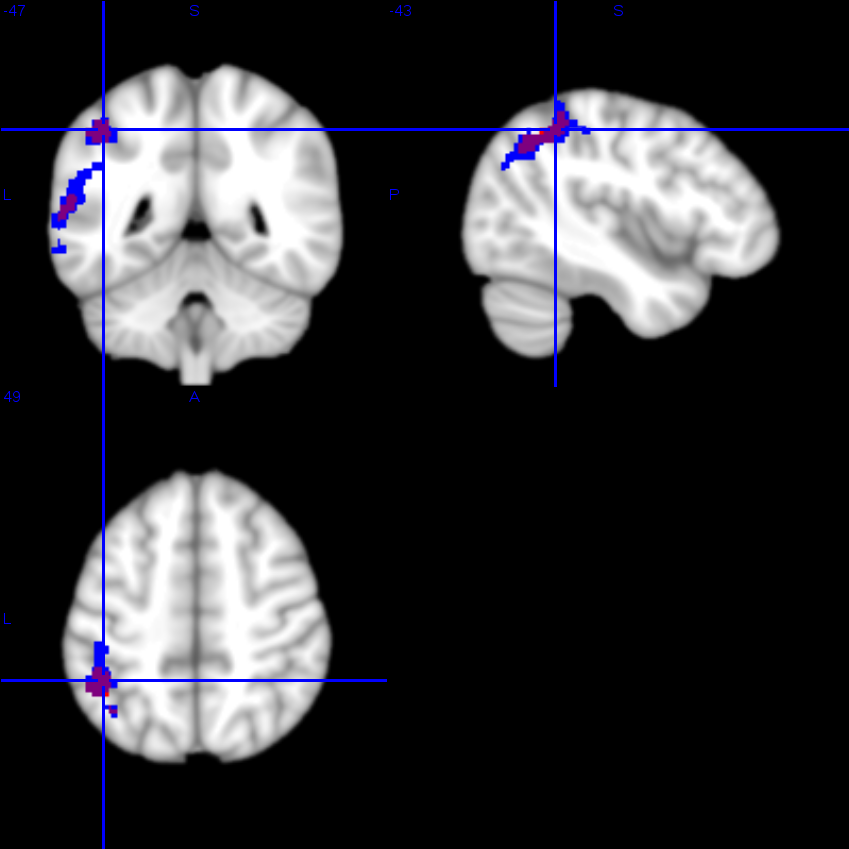

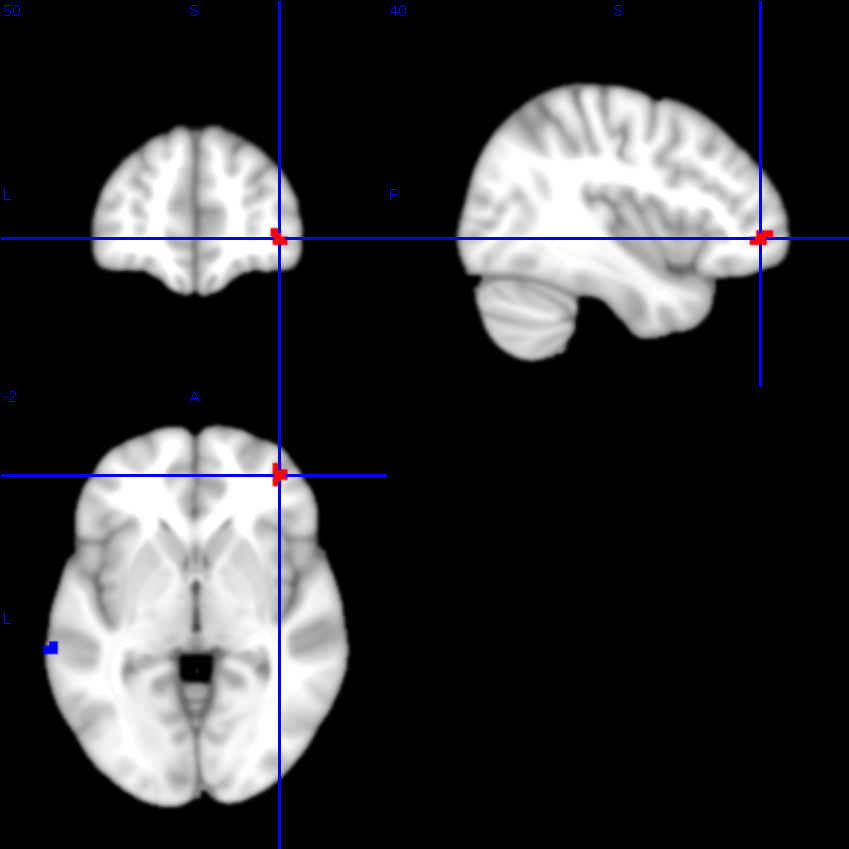


**Figure S1. Regions where significant effects of PEs on R1 were observed after covarying for premorbid IQ (red) and birthweight (blue). Overlap between the two regions is indicated in purple. Other covariates yielded no significant results.**

## 4. Data quality across PE status

To ensure group effects observed were not an artefact due to differences in head motion or mis-registration, we quantified various measures of data quality and checked for significant group effects using independent sample t-tests (binary model) and Spearman correlations (ordinal model).

Head motion was measured in the DWI acquisition by the Euclidian norm of the transform matrix resulting from the rigid body transformations performed during motion correction. Each transform matrix was relative to the mean head position. These mean displacements were compared between groups. Box plots showing the distributions of head motion are shown in figure S2. Spearman correlation between PE status and head movement showed no significant effects (ordinal model: ρ=0.056, p=0.38, binary model: t=0.90, p=0.38).


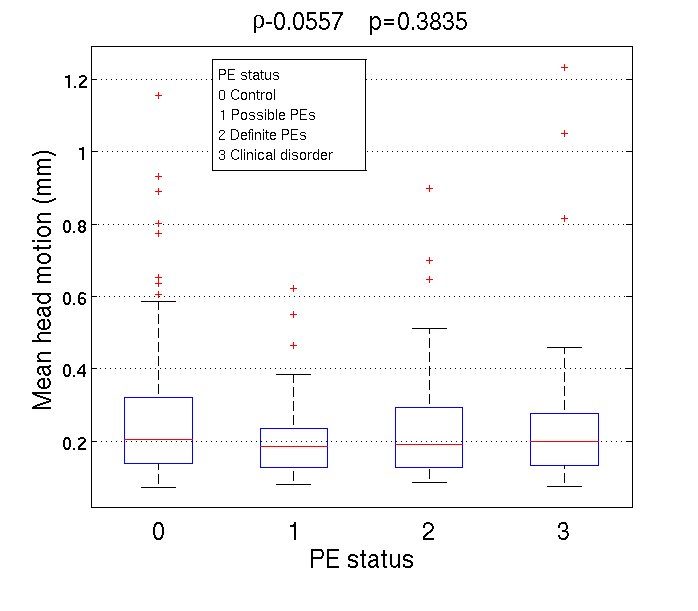


Figure S2. Box plot showing mean displacement of head coordinates during the course of the DWI scan for subjects in each group.

Accuracy of registration between the DESPOT1 and FSPGR images was measured to ensure that group effect in metric derived form these two across groups is not influenced by mis-registration errors. We quantified this in two ways:

Firstly, the magnitude of the transformation from DESPOT1 to the FSPGR space was computed from the norm of the affine transform matrix. Box plots showing the norm of the transform matrices are shown in figure S3. All group effects were non-significant (ordinal model: ρ=0.034, binary mode: p=0.60; t=0.20, p=0.37).


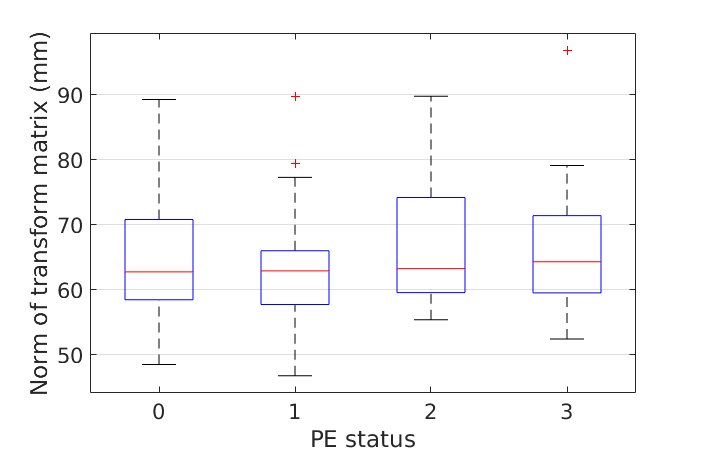


Figure S3. Box plot showing the norm of the transform matrix from DESPOT1 to FSPGR space for subjects in each group.

Secondly, The sum squared error (SSE) was computed between the GM segmentations computed from the high resolution FSPGR T1-wighted image and those computed from the lower resolution synthetic T1-weighted image derived from the DESPOT protocol (as described in the main text).. Box plots showing the norm of the transform matrix are shown in figure S4. No significant group effects were found in the SSE (ordinal model: ρ=-0.075, p=0.23; binary model: t=0.55, p=0.58).


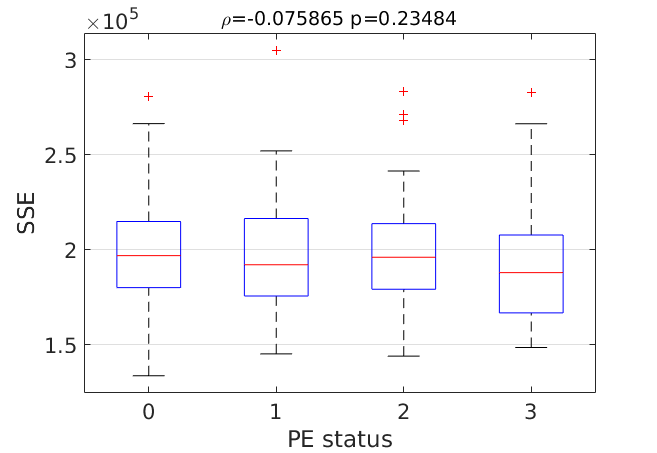


Figure S4. Box plot showing SSE between the GM compartments derived from the FSPGR image and the synthetic T1-weighrted image for subject in each group.

## 5. GM and R1 data at respective site of peak effects

We show here the distribution of data (GM volume and R1) points at the location where GM volume shows peak effect with the binary model (1^st^ column) and where the R1 shows peak effect with the ordinal model (2^nd^ column). The distribution of GM volume in the location of peak R1 effect, although not significant does show a trend towards higher PEs being associated with reduced GM volume. There is no clear trend for R1 in the location of peak GM volume effect.

**Figure S5. Scatter plots of data-points for GM volume and R1 values in the location of peak effect**

|  | Voxel of peak GM vol effect (binary model) | | Voxel of peak R1 effect (ordinal model) |  |
| --- | --- | --- | --- | --- |
| GM VOlume | |  |  |  |
| R1 | |  |  | |
